# Interventions to treat cutaneous leishmaniasis in children: A systematic review

**DOI:** 10.1371/journal.pntd.0006986

**Published:** 2018-12-14

**Authors:** Andrés Uribe-Restrepo, Alexandra Cossio, Mayur M. Desai, Diana Dávalos, María del Mar Castro

**Affiliations:** 1 Departamento de Salud Pública, Universidad Icesi, Cali, Colombia; 2 Unidad Clínica de Leishmaniasis, Centro Internacional de Entrenamiento e Investigaciones Médicas (CIDEIM), Cali, Colombia; 3 Universidad Icesi, Cali, Colombia; 4 Yale School of Public Health, New Haven, CT, United States of America; 5 EDCTP/TDR Fellow. European Vaccine Initiative, UniversitätsKlinikum Heidelberg, Heidelberg, Germany; Insitut Pasteur de Tunis, TUNISIA

## Abstract

**Background:**

Case management in children with cutaneous leishmaniasis (CL) is mainly based on studies performed in adults. We aimed to determine the efficacy and harms of interventions to treat CL in children.

**Methods:**

We conducted a systematic review of clinical trials and cohort studies, assessing treatments of CL in children (≤12 years old). We performed structured searches in PubMed, CENTRAL, LILACS, SciELO, Scopus, the International Clinical Trials Registry Platform (ICTRP), clinicaltrials.gov and Google Scholar. No restrictions regarding ethnicity, country, sex or year of publication were applied. Languages were limited to English, Spanish and Portuguese. Two reviewers screened articles, completed the data extraction and assessment of risk of bias. A qualitative summary of the included studies was performed.

**Results:**

We identified 1092 records, and included 8 manuscripts (6 Randomized Clinical Trials [RCT] and 2 non-randomized studies). Most of the articles excluded in full-text review did not report outcomes separately for children. In American CL (ACL), 5 studies evaluated miltefosine and/or meglumine antimoniate (MA). Their efficacy varied from 68–83% and 17–69%, respectively. In Old-World CL (OWCL), two studies evaluated systemic therapies: rifampicin and MA; and one study assessed efficacy of cryotherapy (42%, Per Protocol [PP]) vs intralesional MA (72%, PP). Few studies (4) provided information on adverse events (AEs) for children, and no serious AEs were reported in participants. Risk of bias was generally low to unclear in ACL studies, and unclear to high in OWCL studies.

**Conclusion:**

Information on efficacy of treatment for CL in children is scarce. There is an unmet need to develop specific formulations, surveillance of AEs, and guidelines both for the management of CL and clinical trials involving the pediatric population.

**Registration:**

The protocol of this review was registered in the PROSPERO International register of systematic reviews, number CRD42017062164.

## Author summary

Cutaneous leishmaniasis (CL) is a parasitic disease that causes chronic, often ulcerated, skin lesions that leave lifelong scars on the face or other visible areas. In some regions of the world, children represent a high proportion of cases. Treatment options for children are limited, and may require administration of poorly tolerated drugs. Despite the differences in responses to these drugs, treatment regimens for children are based on extrapolation of efficacy data in adults. We systematically reviewed the medical literature, searching for controlled studies assessing treatments for CL in children. Eight articles (461 patients aged 2–15 years) were included. None of the studies enrolled children <2 years of age. Identified treatments were miltefosine, systemic and intralesional meglumine antimoniate (MA), cryotherapy, and rifampicin. Sub-optimal quality and small sample sizes limit the generalizability of results from some of these studies. In general, for the Americas, oral miltefosine showed high efficacy, and in an Iranian study, intralesional MA showed higher efficacy than cryotherapy. This study provides evidence of the scarcity of data available to support treatment recommendations in children and of the unmet need to develop and test better treatment options for this vulnerable population.

## Introduction

Cutaneous leishmaniasis is the most common presentation of Leishmaniasis, with global estimates of 0.7 to 1.2 million cases per year[1]. In contexts of peri-domestic and anthroponotic transmission, children represent an important number of cases. In 2016 for the Americas, among 48,915 reported cases of CL, 15.5% were children ≤10 years of age[2]. In the Eastern-Mediterranean region, 100,000 new cases of CL are reported annually[3], and previous reports from Iran note that pediatric patients comprise 7–10% of the cases[4].

Children are a special population of CL cases. Observational studies and clinical trials have shown higher proportions of failure of treatment with first line drugs for CL in children[5–8] compared with adults, especially in younger age groups (<12 years of age)[9]. Differences in immune response[10], drug clearance[11] and overall drug exposure [5,12] in pediatric patients have contributed to this disparity in therapeutic response. Furthermore, the anatomical, physiological and biochemical changes that occur from birth to adolescence affect the pharmacokinetics/pharmacodynamics (PK/PD) of drugs[13] and the frequency and type of adverse events[14].

There are no specific guidelines for the treatment of CL in the pediatric population, and children are generally treated using interventions developed and tested for adults. Considering this information and policy gap, we sought to summarize the literature in terms of efficacy and safety of antileishmanial drugs in children (≤12 years of age).

## Methods

**Study design and literature search:** We conducted a systematic review of the literature following a protocol registered in the PROSPERO International register of systematic reviews, number CRD42017062164. The PICO question[15] covered a **population** of pediatric patients with confirmed diagnosis of cutaneous leishmaniasis without mucosal or visceral involvement; **intervention** with systemic and local treatments for cutaneous leishmaniasis; **comparison group** with placebo, active drug or local treatments. The **outcomes** of interest for this review were proportion of cured patients in each arm and treatment safety (frequency of adverse events). **Study designs** included randomized clinical trials and non-randomized (cohort) studies.

**Inclusion criteria:** Original articles that assess treatments of cutaneous leishmaniasis including pediatric patients (≤12 years of age), with no restrictions regarding ethnicity, country, sex, or year of publication.

**Exclusion criteria** were: 1) articles assessing a disease other than cutaneous leishmaniasis (e.g. mucosal or muco-cutaneous leishmaniasis, visceral leishmaniasis); 2) publication language other than English, Spanish or Portuguese; 3) case reports, case series, case-control studies and systematic reviews/meta-analyses; 4) in vitro or animal studies; 5) full-text not available (after two requests to corresponding authors); 6) reviews, books, and articles without available full texts (conferences, editorials, author responses); 7) articles that did not report outcomes separately for children; and 8) duplicated reports.

**Search strategy and references/data management:** From December 2016 to February 2017, two reviewers (MdMC and AUR) performed an independent literature search. Structured searches were conducted in electronic bibliographic databases: MEDLINE (via PubMed), the Cochrane Central Register of Controlled Trials (CENTRAL), LILACS, SciELO, Scopus, the WHO- International Clinical Trials Registry Platform (ICTRP), clinicaltrials.gov (U.S. National Institutes of Health) and Google Scholar for gray literature. The search terms used were “*cutaneous leishmaniasis*”; “*treatment*”, “*therapy*”, “*management*”; “*outcome*”, “*cure*” or “*failure*”; and “*infant*”, “*child*” or “*adolescent*”. The search strategy used in PubMed is provided as an example: (Cutaneous AND leishman*) AND (treatment OR management OR Therapy) AND (outcome* OR cure OR failure) AND (infant[MeSH] OR child[MeSH] OR adolescent[MeSH]). See supplementary information (S1 File) for a complete list of terms adapted to each search engine’s requirement.

We limited the search to articles with human participants and to three languages: English, Spanish, and Portuguese. No restrictions regarding ethnicity, country, sex, or year of publication were applied in this search strategy. If full-text articles were unavailable, authors were contacted by e-mail (e.g. for unpublished studies) up to a maximum of two attempts within a period of two weeks. Manuscripts whose authors did not answer after the second request were excluded.

Additional search of references included in previously published major systematic reviews of interventions for cutaneous leishmaniasis [16–18] was performed (by AUR) in order to find relevant references that were not captured in the initial search.

Two programs were used to manage references and conduct the review process. The lists of references from different databases and reviewers were imported into Mendeley Reference manager to merge the records and remove duplicates. Thereafter, an overall list of references was imported to the online software Covidence[19] to perform screening by title/abstract, full text review, data extraction and risk of bias assessment.

**Screening and inclusion of studies:** First, the two reviewers performed an independent screening of titles and abstracts identified in the literature searches. Subsequently, the reviewers performed a full text review, applying the a priori inclusion and exclusion criteria. Disagreements in inclusion/exclusion of abstracts and articles were solved by consensus or by a third reviewer (AC).

**Data extraction and summary:** Two reviewers (MdMC and AUR) independently extracted the relevant data on the pediatric population, using a predefined template, based on the PICO question and adapted to the software Covidence[19]. Disagreements between reviewers were resolved by consensus or by a third researcher (AC). Data extracted included: 1) Primary outcome: proportion of patients with therapeutic cure (or therapeutic failure if it was the only outcome reported) in each arm of study, and the definitions of these variables used by the study authors. 2) The secondary outcomes were drug safety (frequency of adverse events) and time to cure (if available). 3) Sociodemographic and clinical data: age range (of pediatric population); total enrolled patients and number of pediatric participants; diagnostic methods and *Leishmania* species. 4) Setting and methodology: country where the study was conducted, aim, design, inclusion/exclusion criteria, intervention/comparator, and follow-up.

We performed an assessment of the risk of bias for each individual study following the Cochrane Collaboration tool[15] and the Newcastle Ottawa Scale[20] for non-randomized studies. A qualitative summary of the evidence was conducted, since a meta-analysis was not planned for this study, considering the expected high heterogeneity of design and outcome definitions, which has been evident in previous systematic reviews[16–18].

## Results

### Characteristics of included studies

**Fig 1:**
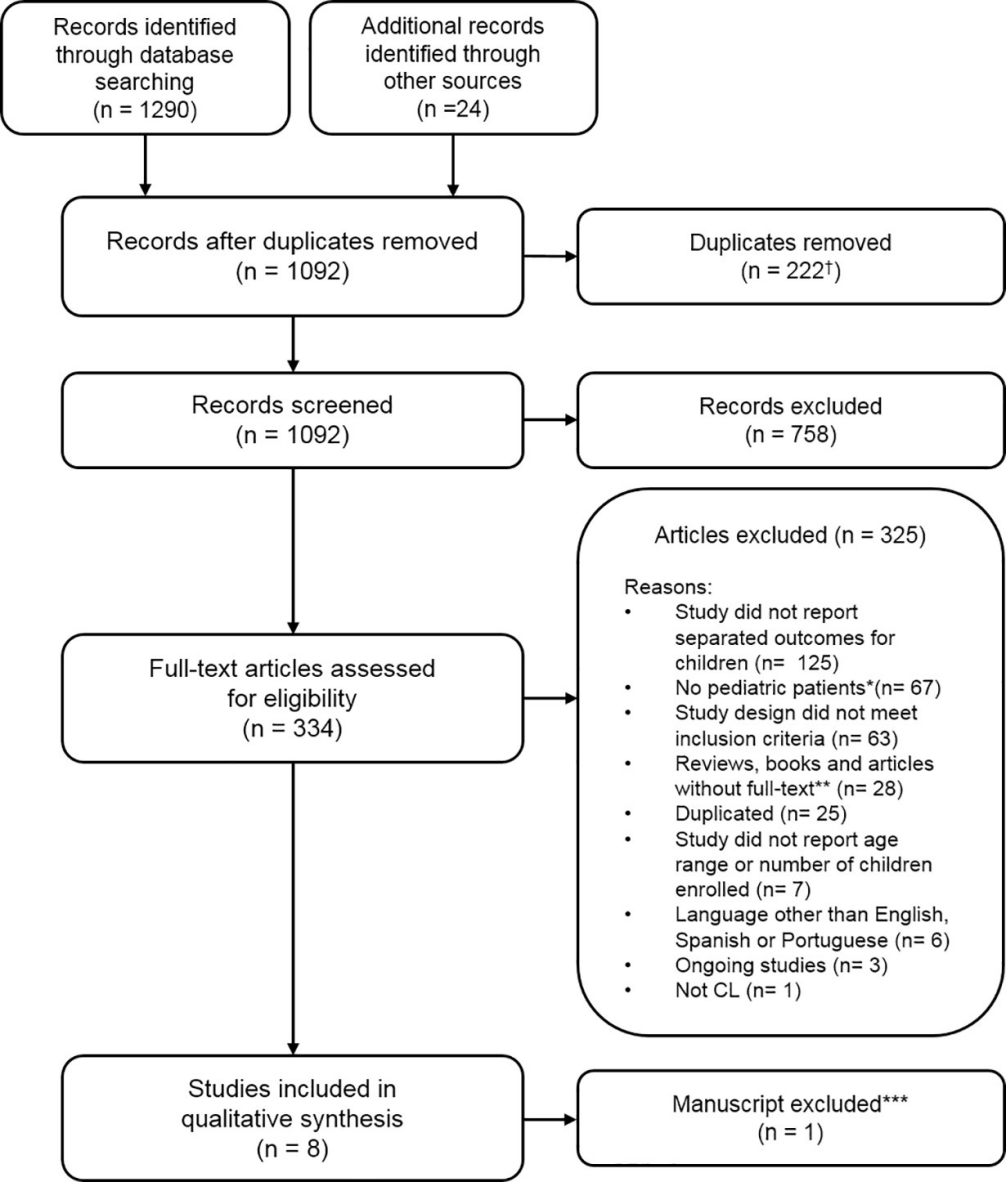
Flow diagram of records identified, screened and included in the review ^†^ n = 219 removed in Mendeley, n = 3 duplicates removed by Covidence. * Children >12 years old. ** Including manuscripts without full-text available, after failed attempts to contact the authors by email (n = 15). *** Considered irrelevant by consensus (see text in “Characteristics of included studies”).

The database searches resulted in 1290 records; 222 duplicates were removed and another 24 reports were added as part of the review of references of other studies (Fig 1). Of the remaining 1092 records, 758 were excluded based on evaluation of title and abstract; 334 full-text articles were evaluated, and nine (9) articles were selected for data extraction. Excluded studies were mostly those that included children and adults but failed to report outcomes separately for pediatric patients (n = 125) or were performed only in adults or adolescents (>12 years old). While reviewing data from the 9 selected studies, one of these[21] was excluded as it tested a therapeutic approach (diminazene aceturate) not currently used in clinical practice. This intervention was previously reported as having insufficient evidence to support its use[16].

The eight included studies were published between 2001 and 2017, principally during the last ten years (6/8 articles). Six were randomized controlled trials, and two were non-randomized studies (Table 1). Five studies were conducted in the Americas[5,7,22–24], and all of them assessed systemic treatments. The 3 studies conducted in Old-World CL endemic countries (Saudi Arabia, Sudan and Iran) assessed both local and systemic therapies[25–27].

**Table 1:** Characteristics of included studies.

Reference Author/year	Country	Study Design	Population	Intervention	Comparator	Outcomes	Duration of follow-up
**ACL**							
**Castro MdM, et al / 2017[5]**	Colombia.	Non-randomized study (Open-label PK clinical trial)	Inclusion criteria: 1) Adults aged 18–60 years and children aged 2–12 years (weight >10 kg); 2) parasitologically confirmed CL; 3) availability for 6 months follow-up after treatment. Exclusion criteria: 1) Mucocutaneous disease; 2) use of antileishmanial drugs during 6 months prior diagnosis; 3) medical history of cardiac, renal, or hepatic disease; 4) menarche (females ≤12 years of age), 5) pregnancy; 6) Baseline AST/ALT, creatinine, BUN or hemoglobin outside normal range.	Miltefosine: Dose: 2.5mg/kg/day Route of administration: oral. Duration: 28 consecutive days.	NA	Primary: Plasma and intracellular PK in children and adults. Secondary: Clinical and parasitological responses; safety (clinical and laboratory adverse events).	210 days after initiation of treatment.
**Chrusciak-T A, et al / 2011[23]**	Brazil.	Randomized Controlled Clinical Trial.	Inclusion criteria: 1) Aged 2–65 years; 2) clinical diagnosis of CL with 1–5 lesions with at least one ulcerated lesion with a diameter of 1–5 cm; 3) time of evolution ≤ 3 months; 4) parasitological confirmation of CL; 5) no previous Leishmania treatment. Exclusion criteria: 1) Evidence of immunodeficiency or antibodies to HIV); 2) pregnancy or unable to use contraceptives during and 3 months after the end of therapy; 3) AST/ALT ≥3xULN, bilirubin ≥2xULN, creatinine and BUN ≥1.5xULN; 4) Evidence of serious underlying disease (cardiac, renal, hepatic or pulmonary) including serious infection other than CL.	Miltefosine: Dose: 2.5mg/kg/day Route of administration: oral. Duration: 28 consecutive days.	Meglumine antimoniate: Dose: 15mg/kg/day. Route of administration: intravenous. Duration: 20 consecutive days.	Primary: Definite cure (at 6 months follow-up visit). Secondary: Safety (clinical and laboratory adverse events)	Six months after end of treatment.
**Machado P, et al / 2010[24]**	Brazil.	Randomized Controlled Clinical Trial.	Inclusion criteria: 1) Aged 2–65 years; 2) ≤ 5 ulcers, with no more than 2 body regions involved; 3) lesion size 10–50 mm in a single dimension; 4) <90 days from the onset of the first ulcer. Exclusion criteria: 1) History of CL or antimony use; 2) patients with evidence of mucosal or disseminated disease; 3) pregnant or breastfeeding women; 4) HIV or any systemic severe disease.	Miltefosine: Dose: 2.5mg/kg/day Route of administration: oral. Duration: 28 consecutive days.	Meglumine antimoniate: Dose: 20mg/kg/day. Route of administration: intravenous. Duration: 20 consecutive days.	Primary: Cure at 6 months after the end of therapy Secondary: Cure at 2 months after the end of therapy, safety (clinical and laboratory adverse events).	Six months after end of therapy.
**Palacios R, et al / 2001[7]**	Colombia.	Randomized Controlled Clinical Trial.	Inclusion criteria: 1) Parasitological diagnosis of CL. Exclusion criteria: 1) Previous treatment with antimonials, ketoconazole or another imidazole, amphotericin B or pentamidine; 2) mucosal leishmaniasis; 3) severe cardiovascular, renal, hepatic, or pancreatic disease; 4) pregnant or nursing women.	Meglumine antimoniate: Dose: 20mg/kg/day. Route of administration: intramuscular. Duration: 10 consecutive days.	Meglumine antimoniate: Dose: 20mg/kg/day. Route of administration: intramuscular. Duration: 20 consecutive days.	Primary: Final clinical response (cure). Secondary: Adverse events (Clinical data).	52 weeks after initiation of treatment.
**Rubiano LC, et al/ 2012[22]**	Colombia.	Randomized Controlled Clinical Trial.	Inclusion criteria: 1) Children aged 2–12 years; 2) parasitologically confirmed CL; 3) patients available to receive supervised treatment for 28 days and follow-up for 26 weeks. Exclusion criteria: 1) Weight <10 kg; 2) mucocutaneous disease; 3) Use of antileishmanials during the month prior diagnosis; 4) history of cardiac, renal, or hepatic disease; 5) menarche; 6) Baseline AST/ALT, hemoglobin, amylase, creatinine and BUN outside the normal range.	Miltefosine: Dose: 2.5mg/kg/day. Route of administration: oral. Duration: 28 consecutive days.	Meglumine antimoniate: Dose: 20mg/kg/day. Route of administration: intramuscular. Duration: 20 consecutive days.	Primary: Therapeutic failure at or before 26 weeks after initiation of treatment. Secondary: Adverse events (clinical and laboratory data), parasitologic response.	26 weeks after initiation of treatment.
**OWCL**							
**Jaffar H/ 2006**[25]	Saudi Arabia.	Randomized Controlled Clinical Trial.	Inclusion criteria: 1) Parasitologically confirmed CL. Exclusion criteria: No data of exclusion criteria.	Rifampicin: Dose:10mg/kg/day. Route of administration: oral. Duration: 4–6 weeks.	Placebo: Dose: No data Route of administration: oral. Duration: 4–6 weeks.	Primary: Time to complete healing of the lesions. Secondary: Effect of treatment in children and adults.	Eight weeks.
**Layegh P, et al / 2009[26]**	Iran.	Randomized Controlled Clinical Trial.	Inclusion criteria: 1) Parasitologically confirmed CL; 2) aged ≤13 years; 3) visited the dermatology clinic September 2006-June 2007; 4) lesions with duration < 12 weeks Exclusion criteria: 1) patients >13 years of age; 2) lesion history >3 months; 3) allergic to antimonial drugs, 4) using simultaneously any other therapeutic method.	Cryotherapy: Dose: -195°C liquid nitrogen applied twice/lesion (20 seconds interval) Route of administration: local Duration: weekly, up to 6 weeks.	Meglumine antimoniate: Dose: 0.5-2cm^3^/lesion. Route of administration: intralesional. Duration: no data	Primary: Definitive cure. Secondary: Time to failure	Six months after the sixth week of treatment.
**Layegh P, et al / 2011[27]**	Iran.	Non-randomized study.	Inclusion criteria: 1) Parasitologically confirmed CL. Exclusion criteria: 1) Lesion history >3 months; 2) allergy to antimoniate compounds; 3) simultaneous use of any other therapeutic methods.	Meglumine antimoniate: Dose: 20mg/kg/day. Route of administration: intramuscular. Duration: 20 consecutive days.	NA	Primary: Complete healing of all skin lesions (cure). Secondary: No reported.	Day 45 after end of treatment.

PK: Pharmacokinetics; AST: Aspartate Aminotransferase; ALT: Alanine Aminotranfserase; BUN: Blood urea nitrogen; CL: Cutaneous Leishmaniasis

The principal inclusion criterion for study participants in the eight articles was parasitological confirmation of CL, and all excluded patients with previous anti-leishmanial treatment, mucosal or muco-cutaneous disease. The number of lesions varied among studies. Length of follow-up was also variable, but in general, it was longer for the studies in the American region (6–12 months) than for those conducted in other regions (45 days to 6 months).

Regarding definition of therapeutic response, in the ACL studies cure was defined as complete re-epithelization of lesions and absence of inflammatory signs such as induration, crust or raised borders. Final response was defined at 6–7 months after initiating treatment in four studies (two studies counted 6 months from the end of treatment[23,24]), and at 12 months in one study[7]. Initial response was determined at 3 months (13 weeks or 2 months after treatment) for most of the studies (Table 2).

**Table 2:** Description of patients and outcomes of treatment in the included studies.

Reference (Author)	Enrolled participants (n)	Enrolled children n (%)	Age of enrolled children (years old; range*)	*Leishmania* species (%)	Definition of cure	Cure in children ITT n/N (%)	Cure in adults ITT n/N (%)	Cure in children PP n/N (%)	Cure in adults PP n/N (%)
**ACL**									
Castro MdM, et al.[5]	60	30 (50)	2–12	*L*.*(V) panamensis* (66.7); *L*.*(V) braziliensis* (3.3); Not isolated (30).	Complete re-epithelialization and absence of inflammatory signs for all lesions at day 210.	**Miltefosine:** 24/30 (80)	**Miltefosine:** 28/30 (93.3)	**Miltefosine:** 24/29 (82.7)	**Miltefosine:** 28/28 (100)
Chrusciak-T A, et al.[23]	90	30 (33.3)	4–12	*L*.*(V) guyanensis* (93.3); *L*.*(V) braziliensis* (3.3); *L*.*(V) lainsoni* (3.3).	Complete epithelialization of all ulcers and complete disappearance of inflammatory induration from all lesions at 6 months follow-up visit.	No data	No data	**Miltefosine:** 12/19 (63.1) **Meglumine antimoniate:** 5/9 (55.5).	**Miltefosine:** 28/37 (75.7) **Meglumine antimoniate:** 10/19 (52.6)
Machado P, et al.[24]	90	32 (35.5)	4–12	*L*.*(V) braziliensis* (45.5).	Lesions with complete re-epithelialization, without raised borders, infiltrations or crusts at 6 months after the end of therapy.	**Miltefosine:** 15/22 (68.2) **Meglumine antimoniate:** 7/10 (70)	**Miltefosine:** 30/38 (78.9) **Meglumine antimoniate:** 9/20 (45).	No data	No data
Palacios R, et al.[7]	136	86 (63.2)	≤14**	*L*.*(V) panamensis* (95.5); *L*.*(V) braziliensis* (4.5) †	Initial clinical response (complete re-epithelialization and absence of inflammatory signs of all lesions at 13 weeks after initiation of treatment) and no relapses of lesions between 13 and 52 weeks of follow-up.	**Meglumine antimoniate** **10 days:** 0–4 y/o: (17) 5–14 y/o: (53) **20 days:** 0–4 y/o: (24) 5–14 y/o: (68)	**Meglumine antimoniate** **10 days:** ≥15 y/o: (61) **20 days:** ≥15 y/o: (69)	**Meglumine Antimoniate** **10 days:** 0–4 y/o: 1/9 (11) 5–14 y/o: 14/21 (67) **20 days:** 0–4 y/o: 2/8 (25) 5–14 y/o: 12/16 (75)	**Meglumine Antimoniate** **10 days:** >14 y/o: 13/16 (81) **20 days:** >14 y/o: 10/12 (83)
Rubiano LC, et al.[22]	116	116 (100)	2–12	*L*.*(V) panamensis* (71.7); *L*.*(V) guyanensis* (26.6); *L*. *(V) braziliensis* (1.7)	Initial clinical therapeutic response (complete re-epithelialization and absence of inflammatory signs of all lesions) attained by week 13 and maintained until week 26 without the appearance of new lesions.	**Miltefosine:** 48/58 (82.8) ^a^ **Meglumine antimoniate:** 40/58 (69) ^a^	Not applicable	**Miltefosine:** 48/55 (87.3) ^a^ **Meglumine antimoniate:** 40/56 (71.4) ^a^	Not applicable
**OWCL**									
Jaffar H. **[25]**	62	32 (51.6)	3–11	Not reported. Author describes *L*.*(L) tropica*. and *L*.*(L) major* as endemic species in the region.	Complete healing of lesions at the end of three months.	No data	No data	**Rifampicin:** 15/18 (83.4) **Placebo:** 2/3 (66.6).	**Rifampicin:** 6/16 (37.5) **Placebo:** 1/4 (25).
Layegh P, et al. 2009[26]	79	79 (100)	**Cryotherapy:** 6.8 (3.4) *** Intralesional MA: 6.2(3.4) ***	Not reported. Authors describe *L*.*(L) tropica* as endemic specie in the region.	Full re-epithelialization of lesions; disappearance of edema, induration, and other signs of inflammation; and a negative direct skin smear result at six months after treatment.	**Cryotherapy:** 15/40 (37.5) ^a^ **Intralesional meglumine antimoniate:** 26/39 (66.7) ^a^	Not applicable	**Cryotherapy:** 15/36 (41.7) ^a^ **Intralesional meglumine antimoniate:** 26/36 (72.3) ^a^	Not applicable
Layegh P, et al. 2011[27]	112	56 (50)	≤15**	Not reported. Authors describe *L*.*(L) tropica* as endemic species in the region.	Full re-epithelialization of ulcers and 100% decrease of induration size for papulo-plaque or nodular lesions, at 45 days after treatment.	**Meglumine antimoniate:** 18/56 (32.1) ^a^	**Meglumine antimoniate:** 31/56 (55.4) ^a^	**Meglumine antimoniate:** 18/51 (35.3) ^a^	**Meglumine antimoniate:** 31/49 (63.3) ^a^

* Data presented as range, unless other measure is specified

** No lower age limit was reported

*** Mean (SD)

† Data available for 88 of 136 patients; y/o: years old

^**a**^ Proportion of cure was calculated by the reviewers, as authors reported treatment failure in the original manuscript.

For OWCL, definitions of therapeutic outcome varied among authors. In the studies by Layegh et al, there is a clear description of criteria of cure (Table 2), including re-epithelization of ulcerated lesions and complete resolution of induration for other type of lesions[26,27]. Jaffar[25] describes the outcome as “complete healing of lesions at the end of three months”. The timing for measuring the outcome also varied in OWCL studies, from 45 days to 6 months, being different for each OWCL study included in the review (Table 1).

### Characteristics of the pediatric patients included in the studies

A combined population of 461 children with CL was included in this review. Reported ages ranged between 2 and 15 years, although two studies did not report the lowest age of enrolled patients[7,27] (Table 2). Distribution by sex was described in five studies [5,22,23,26,27], with the proportion of male patients ranging from 41% to 61%, except for the study by Chrusciak-Talhari et al[23], with a higher proportion of males (65% [13/20] and 70% [7/10]) in both treatment groups. Information of *Leishmania* species was available only for American CL studies and the most frequently isolated species corresponded to *L*.*(V*.*) panamensis*, *L*.*(V*.*) guyanensis* and *L*.*(V*.*) braziliensis* (Table 2). In contrast, OWCL studies did not report isolated *Leishmania* species, and interpretation of efficacy data relies on the known endemic species in the area (Table 1).

### Risk of bias and quality assessment of non-randomized studies

Overall, the risk of bias for the main outcome (therapeutic response) was lower for the studies conducted in the Americas compared to OWCL. Most of the ACL studies reported clearly the random generation of allocation sequence, blinding of main outcome and had a low risk of incomplete outcome reporting. Three of these studies were classified as unclear risk for allocation concealment. In addition, all the clinical trials assessed for ACL were open-label trials, mainly due to the different route of administration of the drugs (oral vs parenteral). Regarding the randomized trials in OWCL, most of the reports were classified as unclear or high risk of bias for all categories within the assessment tool, except for the incomplete outcome data for all outcomes. Detailed risk assessment is available in supplementary Tables 1 and 2 (S1 Table and S2 Table).

One common limitation of the evidence generated in the reviewed studies is the small sample size of each study, and consequent modest generalizability of the results. Only two studies were performed exclusively in children [22,26]. In the remaining articles, efficacy of treatments in pediatric patients was determined by subgroup analysis or as a secondary outcome (as in the study of PK of miltefosine in Colombian patients). These features of the included studies qualify the validity of the findings.

### Articles that did not report outcomes separately for children

Considering the large number of articles excluded, the characteristics of the pediatric population and the age categories used for reporting efficacy data in the excluded articles were described. Seven articles did not report the age range of enrolled participants (Fig 1), and most of them did not report outcomes separately for children <12 years (n = 125). The median sample size of these articles was 85 (IQR: 52–131) and the majority assessed interventions for OWCL (75%, n = 94). Median of the lower age limit of enrolled patients was 5 years old (IQR: 2–7).

Children represented over 50% of the sample in at least 11% of these articles, being as high as 87% in the study of Ben-Salah et al[28] (87% of patients were <18 years old). However, the proportion of children in the sample was not reported in 78% of the articles (n = 97), and the age categories used to describe the population were variable (<18, <15, <12 or <10 years old).

### Interventions evaluated and outcomes of treatment

#### American cutaneous leishmaniasis

**Miltefosine:** Efficacy was assessed in a total of 130 pediatric patients from four studies conducted in the Americas [5,22–24]. The regimen of administration was similar for all studies, with a target dose of 2.5mg/kg/day for 28 days (range 1.5–2.5 mg/kg/day), divided into three daily doses (Table 1). *Leishmania* species represented in the studies of miltefosine were *L*.*(V*.*) panamensis*, *L*.*(V*.*) guyanensis and L*.*(V*.*) braziliensis*. Reported efficacy varied from 63.1% in the smaller studies to 82.8% in the study of Rubiano et al[22], in which the miltefosine treatment arm included the largest number of children with CL evaluated in any study (n = 58); the majority were infected by *L*.*(V*.*) panamensis* (Table 2).

Three publications that included pediatric and adult patients, showed lower efficacy of miltefosine in children (Table 2). However, none of the studies were designed to compare efficacy of miltefosine between these populations, but rather overall efficacy or pharmacokinetics.

In general, studies assessing miltefosine had a low risk of bias for relevant categories, including random generation of the allocation sequence, blinding of outcome measures and incomplete outcome data (S2 and S3). In addition, all the aforementioned studies reported Intention-To-Treat (ITT) analysis, except for Chrusciak-Talhari et al[23], which only described per-protocol (PP) analysis for the subgroup of pediatric patients (Table 2).

**Meglumine antimoniate (local and systemic):** Four studies, with 164 patients evaluated the efficacy of meglumine antimoniate in different doses and routes of administration (Table 1). The most commonly used scheme was 20mg/kg/day IM/IV for 20 days. Some authors, such as Machado et al[24], described a maximum dose of 3 ampoules or 1215mg Sb^V^/day, while others did not report an upper limit of daily dose. Efficacy of Glucantime in ACL studies ranged from 55.5% to 75.0%. However, these values are difficult to compare: the studies by Machado and Chrusciak-Talhari included only 10 pediatric patients each, and the study by Palacios et al[7] stratified the pediatric population as <5 years (efficacy 20-day regimen: 25% PP, 24% ITT) and 5–14 years (efficacy 20-day regimen: 75% PP, 68% ITT) (Table 2). The study with the largest sample was Rubiano et al[22] (n = 58, Glucantime arm), which reported 69% efficacy in a population predominantly infected by *L*.*(V*.*) panamensis*.

When comparing children and adults, the smallest studies presented different results: Chrusciak-Talhari et al[23] showed similar efficacy, and Machado et al[24] found a better response in pediatric patients. However, neither of these studies was powered to estimate differences between children and adults. The largest study reporting children and adults showed lower efficacy in pediatric patients (Table 2). Palacios et al, evidenced substantially lower efficacy in children <5 years of age (p<0.03 for all the comparisons)[7].

#### Old-World cutaneous leishmaniasis

**Meglumine antimoniate:** In total, 95 pediatric patients were included for assessment of this intervention. A non-randomized study reported a 35.3% efficacy of IM Glucantime in patients ≤15 years old, and Layegh et al[26], reported a 72.3% efficacy of intralesional Glucantime in children ≤13 years old (27.7% failure rate, PP). The median number of treatment sessions was 5 (4–6). No isolation of *Leishmania* species was performed in these studies, but authors reported the study setting as endemic for *L*. *tropica and L*. *major*. The latter study was powered to estimate differences of Glucantime vs Cryotherapy in pediatric patients, however, was classified as unclear risk of bias for random generation of the allocation sequence, blinding of outcome measures and allocation concealment.

The single study[27] aimed to compare treatment response in children vs adults reported lower efficacy in children (32.1% vs 55.4%, ITT, p<0.01). This finding is similar to the report by Palacios et al[7] in ACL.

**Cryotherapy with liquid nitrogen:** One study[26] evaluated cryotherapy in children ≤ 13 years old, with a limited efficacy of 41.7% (58.3% failure rate, PP). Treatment was administered weekly, with liquid nitrogen (−195°C) applied twice to the lesion. Each cycle was 10–15 seconds of freezing time with a thawing interval of 20 seconds [25]. The median number of sessions was 4 (3–6). This study was powered and designed to evaluate this therapy in pediatric OWCL, but was classified as having unclear risk of bias for relevant aspects of the Cochrane tool for assessment of risk of bias (S2). Therefore, the evidence for this intervention is considered as unclear risk of bias.

**Rifampicin:** Rifampicin was evaluated for OWCL in a dose of 10mg/kg per day in two equally divided doses during meals for 4–6 weeks in children and adults, with an efficacy of 83.4% (n = 32) in the pediatric population[25], which was higher than the response in adults (37.5%) (n = 30). The author did not report AEs nor parasite species. The setting of the study population is described as endemic for *L*. *major* and *L*. *tropica*, but parasite isolation was not performed.

The study evaluating this treatment has limitations: Regarding the quality assessment, it was classified as unclear risk of bias for the assessment of the outcome. In addition, we consider these results to have limited generalizability because the author did not describe the sample size estimation and losses to follow-up were higher in the placebo group. In addition, it is unclear if efficacy of rifampicin in children was part of a *post hoc* subgroup analysis, given the relatively small sample size of the study (n = 62, with 32 children).

#### Safety (adverse events)

All studies indicated that there were no serious adverse events (such as, hospitalization or death) among study participants. However, only four studies (4/8) reported rates of less severe but common adverse events in children, as other studies presented these data combined with safety data of adults. Two studies compared the frequency of adverse events in children and adults[5,23]. The study by Machado et al[24] found lower frequency of AEs in pediatric patients (66.7% vs 88.3% in adults, p = 0.07) independent of the treatment regimen, while the study by Castro et al[5] did not find significant differences in the type and frequency of adverse events of miltefosine between children and adults.

## Discussion

This systematic review presents a summary of evidence on efficacy and safety for treatments in pediatric populations with cutaneous leishmaniasis. It also provides evidence of the gaps in reporting of treatment outcomes for pediatric patients, even when included in clinical trials. Most articles presented overall results without stratifying outcomes for adult and pediatric populations, and cut-offs for reporting age distribution of participants were variable. Eight reports including 461 pediatric patients aged 2–15 years were reviewed, and no studies enrolled children less than 2 years of age. Miltefosine and meglumine antimoniate were identified as interventions for pediatric ACL, while rifampicin, cryotherapy, systemic and intralesional meglumine antimonate were evaluated for OWCL.

Efficacy of miltefosine and meglumine antimoniate in children varied from 68–83% and 17–69%, respectively. In general, miltefosine shows a higher response rate in the study populations. None of the studies assessed superiority, but the largest reviewed study[22] showed that this drug was non-inferior to meglumine antimoniate in patients with *L*. *panamensis* and *L*. *guyanensis* infection. Thus, for pediatric populations, miltefosine offers a good and achievable therapeutic option, with its oral route of administration, facilitating adherence and enabling home-based supervision of treatment, and access to therapy.

In contrast, for OWCL, evidence was more limited, and the results are difficult to compare due to the variable definitions of therapeutic response, including different length of follow-up. One clinical trial [26] showed that in Iranian children with cutaneous leishmaniasis, intralesional meglumine antimoniate had greater efficacy than cryotherapy (72.3% vs 41.7%, respectively). None of the included studies evaluated the efficacy of miltefosine. One of the excluded studies that did not report outcomes for patients aged <12 years, showed higher efficacy of paromomycin in patients <18 years of age[28]. This intervention was found to have good evidence in previous systematic reviews[16].

Only six studies in this wide literature search assessed efficacy in children and adults separately [5,7,23–25,27]. In general, drug efficacy was lower in pediatric patients compared with adults, independent of the treatment. This is similar to previous studies reporting age as a risk factor for therapeutic failure in leishmaniasis[6,8,29], explained at least partly by differences in pharmacokinetics [5,11,12] and other host factors[10]. However, most studies in CL do not stratify outcomes by age groups or for adults vs. children (125 manuscripts excluded for this reason). In addition, varying age cut-offs for pediatric cases and inconsistent reporting of age categories limit the interpretation of findings regarding treatment outcomes in different age groups and their generalizability.

The role of ontogeny in the disposition and actions of drugs is important to understanding age-related differences in therapeutic response[30], especially in younger children. Efforts to provide a rationale for age subgroups in pediatric trials are ongoing[31] and regulatory guidelines provide some, though arbitrary, reference age categories for pediatric studies[32–34]. Nevertheless, high variability in the boundaries of age categories is common[35], and this is more evident in studies including both children and adults, as described in this review.

Inclusion of children and consideration of the factors influencing drug distribution, metabolism and pharmacokinetics in the design of clinical trials for interventions of CL, and indeed all Neglected Tropical Diseases (NTDs), would improve the quality of evidence supporting treatment recommendations, as well as to provide additional insights on the PK/PD of currently available drugs for CL and other NTDs. Some examples include the allometric dosing of miltefosine for visceral leishmaniasis (NCT02431143), and the dosing of benznidazole for Chagas disease[36].

Importantly, this review also highlights the lack of data regarding treatments in patients less than two years of age, whose treatment options are often considered off-label. Among the current treatment options for leishmaniasis, amphotericin B has evidence of safety in neonates and infants, obtained from clinical trials for systemic fungal infections[37]. However, there is no efficacy data for CL treatment in children under 2 years of age for this or any other recommended treatment. Notably, in a cross-sectional study conducted in a reference center, 9% of pediatric CL cases were less than 2 years old[38]. Off-label use of drugs can be a source of adverse events in children, and other concerns, such as unavailability of appropriate formulations for young children, can affect compliance and effectiveness of drugs[31]. In addition, use of “in house” preparations when no pediatric formulation is available constitutes a risk of dosage error[39]. Patient acceptability, including mode of administration to the child and any related pain or discomfort, are aspects relevant for pharmaceutical development of medicines for pediatric use[32]. These aspects may not be fully met by available drugs for treating CL, where parenteral drugs have been used for decades and oral drugs are only available in capsules, which may be difficult to swallow for young children.

Novel designs in pediatric trials to collect efficacy and PK data, such as opportunistic and scavenged sampling (use of residual blood/plasma from laboratory testing obtained during routine clinical care), are an alternative to address this lack of information and may overcome some of the constraints involved in conducting clinical trials in children[40]. In addition, considering that at least 11% of the articles excluded for not reporting outcomes in children have a sample size formed by more than 50% of pediatric patients, valuable efficacy and safety data could be obtained. Implementation of data sharing platforms and re-analysis of individual-patient data from these studies may overcome the limitations for conducting additional clinical trials in this vulnerable population.

Safety data in children were limited, as they were commonly incorporated with adverse events (AEs) of adults. Reported AEs in the included studies were similar in nature and frequency to those described in adults, although the available data do not allow differences to be fully assessed. Children may be more or less susceptible than adults to the adverse effects of different drugs[14] and some AEs are unique to this population. Monitoring of drug safety in children is critical, because during the process of drug development, clinical trials generate only limited data on AEs in children [41]. Therefore, development and implementation of tools and strategies for surveillance of adverse reactions to antileishmanial treatments, including intralesional and other local therapies in children, warrants attention from the health research community and public health professionals.

Comparison of efficacy data between studies was difficult, in particular for OWCL, due to the differences in outcome definition and duration of follow-up. This variability did not allow a quantitative synthesis of evidence. Previous systematic reviews[16–18] have identified this issue and initiatives to standardize protocols for clinical trials in CL are ongoing[42]. Another limitation of our study is the restriction of the search to three languages (English, Spanish and Portuguese), which might be relevant for OWCL, since articles in other languages that may have valuable information from Africa, the Middle East and Asia were excluded. In our study we defined 12 years of age as the upper limit for inclusion of articles, considering patients >12 years old as adolescents. These age categories are similar to the ICH (The International Council for Harmonization of Technical Requirements for Pharmaceuticals for Human Use) guidelines for pediatric clinical trials[34], but studies using different classifications for pediatric patients (e.g. ≤18 years old), might have been excluded from this analysis.

In conclusion, this study documented the absence of guidelines and scarcity of evidence supporting case management of CL in children. Data sharing platforms to allow individual-patient data analysis, high-quality studies and clinical trials are needed to provide robust data on drug efficacy and safety to support the development of guidelines and implementation of interventions for children with CL.

## Supporting information

S1 File Search strategies used in the electronic databases. (PDF)

S2 File PRISMA checklist. (PDF)

S1 Table Risk of bias assessment for randomized studies. (PDF)

S2 Table Risk of bias assessment for non-randomized studies. (PDF)
