# Supplementary material for: Interventions to treat cutaneous leishmaniasis in children: A systematic review
Source: PLoS Negl Trop Dis. 2018 Dec 14;12(12):e0006986. doi: 10.1371/journal.pntd.0006986 (PMC6310290; doi:10.1371/journal.pntd.0006986)
Supplement: S1 File — (PDF) [file pntd.0006986.s001.pdf]

## S1 File. Search strategies used in the electronic databases.

1. **PubMed:** (Cutaneous AND leishman*) AND (treatment OR management OR Therapy) AND (outcome* OR cure OR failure) AND (infant [MeSH] OR child[MeSH] OR adolescent[MeSH]).

2. **Cochrane Central Register of Controlled Trials**(Cutaneous and leishman*) and (treatment or management or Therapy) and (outcome* or cure or failure) and (infant or child or adolescent) in Trials in the strategy currently being edited

3. **LILACS:** (tw:(cutaneous AND leishman*)) AND (tw:(therapy OR treatment OR management)) AND (tw:(outcome OR cure OR failure)) AND (tw:(pediatric OR children OR adolescent)) AND (instance:"regional") AND (instance:"regional") AND (db:("LILACS" OR "MedCarib") AND la:("en" OR "pt" OR "es"))

4. **Scopus:** (TITLE-ABS-KEY (cutaneous AND leishman*) AND TITLE-ABS-KEY (treatment OR management OR therapy) ANDTITLE-ABS-KEY (outcome* OR cure OR failure) AND TITLE-ABS-KEY (infant OR child OR adolescent)) AND (LIMIT-TO (DOCTYPE, "ar")) AND (LIMIT-TO (SUBJAREA, "MEDI")) AND (LIMIT-TO (LANGUAGE, "English") OR LIMIT-TO (LANGUAGE, "Portuguese") OR LIMIT-TO (LANGUAGE, "Spanish"))

5. **SciELO:** ((cutaneous) AND (Leishman*)) AND ((management) OR (therapy) OR (treatment)) AND ((adolescent) OR (children) OR (infant)).

6. **Web of Science:** (Cutaneous AND leishman* AND ('treatment'/exp OR treatment OR 'management'/exp OR management OR 'therapy'/exp OR therapy) AND (outcome* OR 'cure'/exp OR cure OR 'failure'/exp OR failure) AND ('infant'/exp OR infant OR 'child'/exp OR child OR 'adolescent'/exp OR adolescent)) Timespan: All years.

7. **Clinical Trials:** cutaneous leishmaniasis | cutaneous | Child

8. **Google Scholar (for gray literature): With all of the words:** leishmaniasis or leishmania and children. **With exact phrase:** clinical trial or clinical trials or randomized or randomised or trial or cohort or control. **With at least one of the words:** treatment therapy efficacy therapeutics management. **Without the words:** visceral mucosal mucocutaneous case series reports murine mice dogs canine vitro polymerase IL Polymorphism immune vector. **Complete search:** leishmaniasis or leishmania and children treatment OR therapy OR efficacy OR therapeutics OR management "cutaneous leishmaniasis" -visceral -mucosa -mucocutaneous -case -series -reports -murine -mice -dogs -canine -vitro -polymerase -IL -Polymorphism –vector.
