# Supplementary material for: Interventions to treat cutaneous leishmaniasis in children: A systematic review
Source: PLoS Negl Trop Dis. 2018 Dec 14;12(12):e0006986. doi: 10.1371/journal.pntd.0006986 (PMC6310290; doi:10.1371/journal.pntd.0006986)
Supplement: S1 Table — (PDF) [file pntd.0006986.s003.pdf]

S1 Table. Supplementary table 1. Risk of Bias assessment for randomized studies.

Type of CL / Study	Random generation of allocation sequence (selection bias)	Allocation concealment (selection bias)	Blinding of participants and personnel for all outcomes (performance bias)	Blinding of outcome assessment for main outcome (detection bias)	Incomplete outcome data for all outcomes	Selective reporting of outcomes	Other source of bias
**ACL**							
**Castro MdM, et al.**	Not applicable for this study design	Not applicable for this study design	Not applicable for this study design	Not applicable for this study design	**Low:** Low proportion of lost to follow-up in both groups.	**Low:** Protocol registered in clinicaltrials.gov, NCT01462500	**Unclear:** Study design to assess PK of miltefosine, not powered to assess efficacy.
**Chrusiack-T A, et al.**	**Low:** used StataCorp LP 9 for randomization sequence.	**Unclear:** not described in the manuscript.	**High:** open label trial. Evaluators or participants were not masked.	**Unclear:** for the secondary outcome (Adverse events).	**Low:** Low proportion of patients lost to follow-up or excluded from the analysis.	**Low:** Protocol registered in clinicaltrials.gov, NCT00600548.	**Unclear:** Results for children estimated as part of a subgroup analysis, not considered in the sample size calculation.
**Machado P, et al.**	**Low:** Used a computer-based randomization table	**Unclear:** not described.	**High:** Participants knew intervention assignment due to the characteristics of the drugs (parenteral vs oral administration).	**Low:** Two clinicians masked for the study intervention evaluation.	**Low:** Low proportion of lost to follow-up.	**Low:** Protocol registered in clinicaltrials.gov, NCT00600548.	**Unclear:** Results for children estimated as part of a subgroup analysis, not considered in the sample size calculation.
**Palacios R, et al.**	**Low:** Used permuted block randomization.	**Unclear:** Method not described.	**High:** Blinding not possible, due to ethical considerations.	**Low:** Masking assessors with respect to the study group.	**High:** High proportion of non-adherence and lost to follow-up: 41%	**Unclear:** Study protocol is unavailable.	**Unclear:** Results for children estimated as part of a subgroup analysis, not considered in the sample size calculation.
**Rubiano LC, et al.**	**Low:** Computerized balanced block randomization scheme.	**Low:** Coordinating center via phone call.	**High:** Open label trial. Authors acknowledge the ethical issues of using placebo in this study.	**Low:** Assessors were blinded of the study intervention.	**Low:** Low proportion of lost to follow-up/withdrawals (only 2 and 3 per group) in the study.	**Low:** Protocol registered in clinicaltrials.gov, NCT00487253.	**Low.**
**OWCL**							

**Jaffar H.**	**Unclear:** No details regarding the sequence generation process.	**Unclear:** no described how they did the allocation concealment.	**Unclear:** Double-blind study, although there is a low risk of unblinding, due to the color in the urine associated with the use of rifampicin.	**Unclear:** **D**escribed as double-blind, but there are no details of blinding of the outcome assessors.	**High:** A higher frequency of lost to follow-up in the control group, compared to the intervention (Follow-up: 73.9% vs 43.7%).	**Unclear:** Study protocol is unavailable.	**High:** Sample size were not described. It is unclear if this sample size would allow to make inferences of the results in pediatric patients.
**Layegh P, et al. 2009**	**Unclear:** do not describe the sequence generation process. Only described the study as "randomized".	**Unclear:** no described how they did the allocation concealment.	**High:** No blinding, due to the characteristics of the interventions.	**Unclear:** not described if the outcome assessors were blinded.	**Low:** Low proportion of loss to follow-up.	**Unclear:** Analysis presented as ITT and PP. The protocol with the original outcome variables is not available.	**Low.**
**Layegh P, et al. 2011**	Not applicable for this study design	Not applicable for this study design	Not applicable for this study design	Not applicable for this study design	**Low:** Low proportion of loss to follow-up.	**High:** Protocol is unavailable. Authors report the proportion of failures, a different outcome according to description in the methods (response rate, and as endpoint the definitive cure or withdrawal from the study).	**Low:** Both exposure groups have similar demographic and clinical characteristics, and the proportion of non-adherence was low.
