# Supplementary material for: Interventions to treat cutaneous leishmaniasis in children: A systematic review
Source: PLoS Negl Trop Dis. 2018 Dec 14;12(12):e0006986. doi: 10.1371/journal.pntd.0006986 (PMC6310290; doi:10.1371/journal.pntd.0006986)
Supplement: S2 Table — (PDF) [file pntd.0006986.s004.pdf]

S2 Table.

Supplementary table 2. Risk of bias assessment for non-randomized studies.

Study ref.	Selection				Comparability	Outcome			NOS quality score (num. Of stars)
	Representativeness of exposed cohort	Selection of non-exposed cohort	Ascertainment of exposure	Outcome of interest was not present at start of study	Comparability of cohorts on the basis of the design or analysis controlled for confounders	Assessment of outcome	follow-up long enough for outcomes to occur	Adequacy of follow-up of cohorts	
**Castro MdM, et al**	*	* Controls (adults) drawn from the same community as the exposed cohort	* Intervention (miltefosine) assigned and supervised	*	*	*	*	* 5% lost to follow-up	**8** **Good quality**
**Layegh P, et al 2011**	*	* Controls (adults) drawn from the same community as the exposed cohort	* Intervention (meglumine antimoniate) assigned and supervised	*	*	*	Short follow-up (45 days)	* 10.7% lost to follow-up	**7** **Good quality**

Thresholds for converting the Newcastle-Ottawa scales to AHRQ standards (good, fair, and poor):
Good quality: 3 or 4 stars in selection domain AND 1 or 2 stars in comparability domain AND 2 or 3 stars in outcome/exposure domain
Fair quality: 2 stars in selection domain AND 1 or 2 stars in comparability domain AND 2 or 3 stars in outcome/exposure domain
Poor quality: 0 or 1 star in selection domain OR 0 stars in comparability domain OR 0 or 1 stars in outcome/exposure domain
